# Bis(4′-hy­droxy­biphenyl-4-carboxyl­ato-κ*O* ^1^)(1,10-phenanthroline-κ^2^ *N*,*N*′)zinc

**DOI:** 10.1107/S1600536811012244

**Published:** 2011-04-13

**Authors:** Wei-Ping Wu, Jun Wang, Lu Lu, Xi-Yang He, Li-Ke Zou

**Affiliations:** a School of Chemistry and Pharmaceutical Engineering, Institute of Functionalized Materials, Sichuan University of Science and Engineering, Zigong, Sichuan 643000, People’s Republic of China

## Abstract

In the title compound, [Zn(C_13_H_9_O_3_)_2_(C_12_H_8_N_2_)], the Zn^II^ atom is located on a twofold rotation axis and has a distorted tetra­hedral coordination with two N atoms from the phenanthroline ligand arranged around the twofold axis and two O atoms from two symmetry-related 4′-hy­droxy­biphenyl-4-carboxyl­ate ligands. The mol­ecules are linked by O—H⋯O hydrogen bonds, forming a chain developing parallel to [101].

## Related literature

For background to crystal engineering, see: Aakeroy & Seddon (1993 ▶). For the related carb­oxy­lic acid, see: Song *et al.* (2004 ▶); Liu *et al.* (2011*a* ▶). For the related phenanthroline and its derivative complexes, see: Breneman *et al.* (1993 ▶); Liu *et al.* (2011*b* ▶); Zhang *et al.* (2011 ▶).
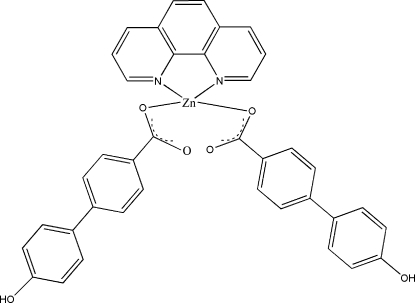


## Experimental

### 

#### Crystal data

[Zn(C_13_H_9_O_3_)_2_(C_12_H_8_N_2_)]
*M* *_r_* = 671.98
Monoclinic, 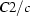

*a* = 15.378 (8) Å
*b* = 10.616 (5) Å
*c* = 17.816 (9) Å
β = 90.702 (9)°
*V* = 2908 (2) Å^3^
*Z* = 4
Mo *K*α radiation
μ = 0.90 mm^−1^
*T* = 298 K
0.30 × 0.27 × 0.21 mm

#### Data collection

Bruker APEXII area-detector diffractometer
Absorption correction: multi-scan (*SADABS*; Bruker, 2008 ▶) *T*_min_ = 0.774, *T*_max_ = 0.833
10508 measured reflections
2605 independent reflections
2273 reflections with *I* > 2σ(*I*)
*R*_int_ = 0.027

#### Refinement

*R*[*F* ^2^ > 2σ(*F* ^2^)] = 0.030
*wR*(*F* ^2^) = 0.092
*S* = 1.06
2605 reflections
214 parameters
H-atom parameters constrained
Δρ_max_ = 0.26 e Å^−3^
Δρ_min_ = −0.32 e Å^−3^

### 

Data collection: *APEX2* (Bruker, 2008 ▶); cell refinement: *SAINT* (Bruker, 2008 ▶); data reduction: *SAINT*; program(s) used to solve structure: *SHELXS97* (Sheldrick, 2008 ▶); program(s) used to refine structure: *SHELXL97* (Sheldrick, 2008 ▶); molecular graphics: *ORTEPIII* (Burnett & Johnson, 1996 ▶), *ORTEP-3 for Windows* (Farrugia, 1997 ▶) and *PLATON* (Spek, 2009 ▶); software used to prepare material for publication: *SHELXL97*.

## Supplementary Material

Crystal structure: contains datablocks I, global. DOI: 10.1107/S1600536811012244/dn2671sup1.cif

Structure factors: contains datablocks I. DOI: 10.1107/S1600536811012244/dn2671Isup2.hkl

Additional supplementary materials:  crystallographic information; 3D view; checkCIF report

## Figures and Tables

**Table 1:** Hydrogen-bond geometry (Å, °)

*D*—H⋯*A*	*D*—H	H⋯*A*	*D*⋯*A*	*D*—H⋯*A*
O3—H3*A*⋯O2^i^	0.82	1.81	2.622 (2)	173

Symmetry code: (i) 
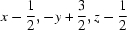
.

**Fig. 1.**
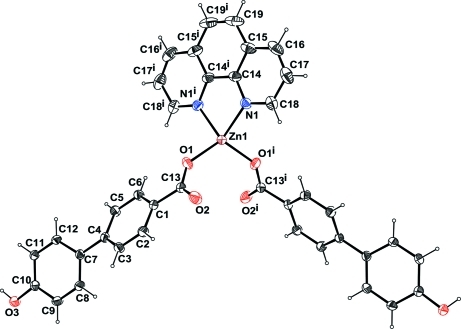
Molecular structure of (I), showing the atom labelling scheme. Ellipsoids are drawn at the 30% probability level. H atoms are represented as small sphere of arbitrary radii. [Symmetry code: (i) -x+1, y, -z+1/2]

**Fig. 2.**
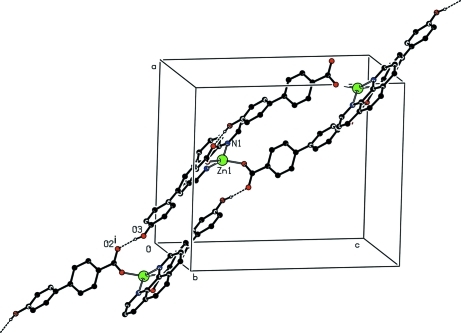
Partial packing view showing the formation of the chain through O-H···O hydrogen bonds which are shown as dashed lines. H atoms not involved in hydrogen bondings have been omitted for clarity. [Symmetry code: (i) x-1/2, -y+3/2, z-1/2]

## supplementary crystallographic information

### Comment

In the past years, many supramolecular motifs based on hydrogen bonds have been achieved by using transition metal centers and organic ligands (Aakeroy *et al.*, 1993). The 4'-hydroxybiphenyl-4-carboxylic acid (H_2_L) has inspired great research interest for assembling coordination architectures. As a versatile ligand, it contains two sulfonic groups and two hydroxyl groups, which may be partially or completely deprotonated and normally serves as linkage to construct diverse metallosupramolecular systems (Song *et al.*, 2004; Liu *et al.*, 2011*a*). On the other hand, 1, 10-Phenanthroline, as one kind of those ligand, has usually been used to construct a great variety of structurally interesting entities, such as monomers(Breneman *et al.* 1993; Liu *et al.*, 2011*b*, Zhang *et al.*, 2011). Herein, we are interested in self-assemblies of Zn^II^ ion with H_2_L and phen, which led to the title compound.

The title compound, {[Zn(L)_2_(phen)] (H_2_L=4'-hydroxybiphenyl-4-carboxylic acid), is built up from a distorted tetrahedral Zn^II ^located on a two fold axis and surrounded by two O atoms of two 4'-hydroxybiphenyl-4-carboxylate ligands and the two N atoms of the phenanthroline ligand (Fig. 1).

The molecules are linked by O-H···O hydrogen bonds, forming a one-dimensional chain parallel to the [1 0 1] direction (Fig. 2, Table 1).

### Experimental

A mixture of Zn(AC)2.H2O(25mg, 0.1mmol), H_2_L(26mg, 0.1mmol), phen(19mg, 0.1mmol), NaOH(0.1mmol) and 5mL H_2_O and CH_3_OH(2mL) was stirred for 3h, and then the mixture was transferred to an 25mL Teflon-lined reactor and kept under autogenous pressure at 423k for 3 days,then cooled down to room temperature. Single crystals suitable for X-ray diffraction were obtained.

### Refinement

All H atoms attached to C and O (hydroxyl group) atoms were fixed geometrically and treated as riding with C—H = 0.93 Å and O—H = 0.82 Å with U_iso_(H) = 1.2U_eq_(C, O).

### Crystal data

**Table:**

[Zn(C_13_H_9_O_3_)_2_(C_12_H_8_N_2_)]	*F*(000) = 1384
*M**_r_* = 671.98	*D*_x_ = 1.535 Mg m^−^^3^
Monoclinic, *C*2/*c*	Mo *K*α radiation, λ = 0.71073 Å
Hall symbol: -C 2yc	Cell parameters from 2606 reflections
*a* = 15.378 (8) Å	θ = 2.3–25.2°
*b* = 10.616 (5) Å	µ = 0.90 mm^−^^1^
*c* = 17.816 (9) Å	*T* = 298 K
β = 90.702 (9)°	Block, colourless
*V* = 2908 (2) Å^3^	0.30 × 0.27 × 0.21 mm
*Z* = 4	

### Data collection

**Table:**

Bruker APEXII area-detector diffractometer	2605 independent reflections
Radiation source: fine-focus sealed tube	2273 reflections with *I* > 2σ(*I*)
graphite	*R*_int_ = 0.027
φ and ω scans	θ_max_ = 25.2°, θ_min_ = 2.3°
Absorption correction: multi-scan (*SADABS*; Bruker, 2008)	*h* = −18→18
*T*_min_ = 0.774, *T*_max_ = 0.833	*k* = 0→12
10508 measured reflections	*l* = 0→21

### Refinement

**Table:**

Refinement on *F*^2^	Primary atom site location: structure-invariant direct methods
Least-squares matrix: full	Secondary atom site location: difference Fourier map
*R*[*F*^2^ > 2σ(*F*^2^)] = 0.030	Hydrogen site location: inferred from neighbouring sites
*wR*(*F*^2^) = 0.092	H-atom parameters constrained
*S* = 1.06	*w* = 1/[σ^2^(*F*_o_^2^) + (0.0557*P*)^2^ + 1.3191*P*] where *P* = (*F*_o_^2^ + 2*F*_c_^2^)/3
2605 reflections	(Δ/σ)_max_ = 0.001
214 parameters	Δρ_max_ = 0.26 e Å^−^^3^
0 restraints	Δρ_min_ = −0.32 e Å^−^^3^

### Special details

**Table:**

Geometry. All esds (except the esd in the dihedral angle between two l.s. planes) are estimated using the full covariance matrix. The cell esds are taken into account individually in the estimation of esds in distances, angles and torsion angles; correlations between esds in cell parameters are only used when they are defined by crystal symmetry. An approximate (isotropic) treatment of cell esds is used for estimating esds involving l.s. planes.
Refinement. Refinement of F^2^ against ALL reflections. The weighted R-factor wR and goodness of fit S are based on F^2^, conventional R-factors R are based on F, with F set to zero for negative F^2^. The threshold expression of F^2^ > 2sigma(F^2^) is used only for calculating R-factors(gt) etc. and is not relevant to the choice of reflections for refinement. R-factors based on F^2^ are statistically about twice as large as those based on F, and R- factors based on ALL data will be even larger.

### Fractional atomic coordinates and isotropic or equivalent isotropic displacement parameters (Å^2^)

**Table:**

	*x*	*y*	*z*	*U*_iso_*/*U*_eq_	
Zn1	0.5000	0.35249 (3)	0.2500	0.04222 (15)	
O1	0.50786 (11)	0.45044 (15)	0.16187 (8)	0.0515 (4)	
O2	0.61479 (12)	0.59120 (16)	0.16290 (10)	0.0650 (5)	
O3	0.20326 (10)	1.04044 (13)	−0.23778 (9)	0.0462 (4)	
H3A	0.1793	0.9948	−0.2689	0.069*	
N1	0.56738 (11)	0.20478 (17)	0.29840 (9)	0.0419 (4)	
C1	0.49614 (12)	0.62166 (18)	0.07931 (10)	0.0338 (4)	
C2	0.53096 (13)	0.73018 (19)	0.04826 (12)	0.0396 (5)	
H2	0.5858	0.7577	0.0637	0.047*	
C3	0.48521 (12)	0.79735 (19)	−0.00498 (11)	0.0380 (4)	
H3	0.5096	0.8700	−0.0249	0.046*	
C4	0.40291 (12)	0.75884 (18)	−0.02979 (10)	0.0313 (4)	
C5	0.36906 (12)	0.64957 (18)	0.00116 (12)	0.0373 (5)	
H5	0.3145	0.6214	−0.0145	0.045*	
C6	0.41457 (13)	0.58227 (19)	0.05454 (11)	0.0382 (5)	
H6	0.3904	0.5094	0.0744	0.046*	
C7	0.35300 (12)	0.83181 (18)	−0.08651 (10)	0.0317 (4)	
C8	0.35415 (13)	0.96324 (18)	−0.08772 (11)	0.0392 (5)	
H8	0.3889	1.0062	−0.0531	0.047*	
C9	0.30532 (13)	1.03091 (19)	−0.13874 (12)	0.0406 (5)	
H9	0.3080	1.1184	−0.1387	0.049*	
C10	0.25204 (12)	0.96926 (18)	−0.19028 (11)	0.0346 (4)	
C11	0.25167 (12)	0.83874 (18)	−0.19150 (11)	0.0349 (4)	
H11	0.2178	0.7961	−0.2269	0.042*	
C12	0.30139 (12)	0.77208 (18)	−0.14041 (10)	0.0340 (4)	
H12	0.3004	0.6845	−0.1420	0.041*	
C13	0.54446 (14)	0.55089 (19)	0.13953 (11)	0.0388 (5)	
C14	0.53520 (14)	0.09192 (19)	0.27718 (12)	0.0423 (5)	
C15	0.56745 (16)	−0.0227 (2)	0.30488 (15)	0.0569 (7)	
C16	0.63435 (18)	−0.0157 (3)	0.35882 (16)	0.0687 (8)	
H16	0.6568	−0.0889	0.3801	0.082*	
C17	0.66621 (18)	0.0979 (3)	0.37981 (15)	0.0672 (8)	
H17	0.7113	0.1027	0.4149	0.081*	
C18	0.63158 (15)	0.2074 (3)	0.34886 (13)	0.0533 (6)	
H18	0.6541	0.2848	0.3640	0.064*	
C19	0.53160 (19)	−0.1375 (2)	0.27629 (18)	0.0741 (9)	
H19	0.5525	−0.2139	0.2945	0.089*	

### Atomic displacement parameters (Å^2^)

**Table:**

	*U*^11^	*U*^22^	*U*^33^	*U*^12^	*U*^13^	*U*^23^
Zn1	0.0656 (3)	0.0293 (2)	0.0316 (2)	0.000	−0.00514 (15)	0.000
O1	0.0671 (10)	0.0459 (9)	0.0411 (9)	−0.0079 (8)	−0.0141 (7)	0.0139 (7)
O2	0.0700 (11)	0.0529 (10)	0.0711 (12)	−0.0120 (8)	−0.0427 (9)	0.0165 (9)
O3	0.0560 (9)	0.0363 (8)	0.0458 (9)	0.0032 (7)	−0.0224 (7)	0.0020 (6)
N1	0.0476 (10)	0.0396 (10)	0.0387 (9)	0.0014 (8)	0.0050 (8)	0.0047 (8)
C1	0.0414 (10)	0.0318 (9)	0.0279 (10)	−0.0009 (8)	−0.0060 (8)	−0.0014 (8)
C2	0.0381 (10)	0.0369 (11)	0.0433 (11)	−0.0089 (8)	−0.0153 (9)	0.0021 (9)
C3	0.0393 (10)	0.0346 (10)	0.0399 (11)	−0.0099 (8)	−0.0079 (8)	0.0053 (9)
C4	0.0334 (9)	0.0331 (10)	0.0274 (9)	−0.0019 (7)	−0.0027 (7)	−0.0017 (8)
C5	0.0332 (10)	0.0401 (11)	0.0384 (11)	−0.0093 (8)	−0.0070 (8)	0.0037 (8)
C6	0.0417 (10)	0.0358 (11)	0.0369 (11)	−0.0094 (8)	−0.0014 (8)	0.0051 (9)
C7	0.0322 (9)	0.0335 (10)	0.0293 (9)	−0.0039 (7)	−0.0030 (7)	0.0007 (8)
C8	0.0450 (11)	0.0328 (10)	0.0395 (11)	−0.0068 (8)	−0.0151 (9)	−0.0028 (8)
C9	0.0472 (11)	0.0278 (10)	0.0464 (12)	−0.0025 (8)	−0.0127 (9)	−0.0008 (8)
C10	0.0353 (9)	0.0362 (10)	0.0321 (10)	0.0009 (8)	−0.0045 (8)	0.0013 (8)
C11	0.0372 (10)	0.0350 (10)	0.0323 (10)	−0.0047 (8)	−0.0088 (8)	−0.0038 (8)
C12	0.0381 (10)	0.0283 (10)	0.0355 (10)	−0.0047 (8)	−0.0043 (8)	−0.0016 (8)
C13	0.0508 (12)	0.0327 (10)	0.0328 (10)	0.0023 (9)	−0.0095 (9)	−0.0013 (8)
C14	0.0490 (12)	0.0332 (11)	0.0453 (12)	0.0032 (9)	0.0177 (9)	0.0044 (9)
C15	0.0637 (15)	0.0422 (13)	0.0657 (16)	0.0161 (11)	0.0328 (13)	0.0134 (11)
C16	0.0699 (17)	0.0684 (18)	0.0684 (18)	0.0340 (15)	0.0241 (14)	0.0281 (15)
C17	0.0565 (15)	0.090 (2)	0.0555 (16)	0.0228 (15)	0.0038 (12)	0.0197 (15)
C18	0.0527 (13)	0.0625 (15)	0.0448 (13)	0.0041 (11)	0.0010 (10)	0.0089 (11)
C19	0.088 (2)	0.0331 (12)	0.103 (3)	0.0104 (11)	0.0473 (17)	0.0107 (13)

### Geometric parameters (Å, °)

**Table:**

Zn1—O1^i^	1.8884 (16)	C7—C12	1.391 (2)
Zn1—O1	1.8884 (16)	C7—C8	1.396 (3)
Zn1—N1^i^	2.0626 (19)	C8—C9	1.375 (3)
Zn1—N1	2.0626 (19)	C8—H8	0.9300
O1—C13	1.272 (3)	C9—C10	1.387 (3)
O2—C13	1.231 (3)	C9—H9	0.9300
O3—C10	1.354 (2)	C10—C11	1.386 (3)
O3—H3A	0.8200	C11—C12	1.377 (3)
N1—C18	1.327 (3)	C11—H11	0.9300
N1—C14	1.349 (3)	C12—H12	0.9300
C1—C2	1.388 (3)	C14—C15	1.401 (3)
C1—C6	1.389 (3)	C14—C14^i^	1.444 (5)
C1—C13	1.499 (3)	C15—C16	1.401 (4)
C2—C3	1.374 (3)	C15—C19	1.429 (4)
C2—H2	0.9300	C16—C17	1.352 (4)
C3—C4	1.397 (3)	C16—H16	0.9300
C3—H3	0.9300	C17—C18	1.390 (4)
C4—C5	1.388 (3)	C17—H17	0.9300
C4—C7	1.480 (3)	C18—H18	0.9300
C5—C6	1.374 (3)	C19—C19^i^	1.342 (7)
C5—H5	0.9300	C19—H19	0.9300
C6—H6	0.9300		
			
O1^i^—Zn1—O1	113.17 (11)	C7—C8—H8	119.1
O1^i^—Zn1—N1^i^	136.83 (7)	C8—C9—C10	120.30 (19)
O1—Zn1—N1^i^	96.20 (7)	C8—C9—H9	119.8
O1^i^—Zn1—N1	96.20 (7)	C10—C9—H9	119.8
O1—Zn1—N1	136.83 (7)	O3—C10—C11	123.09 (17)
N1^i^—Zn1—N1	81.03 (10)	O3—C10—C9	117.94 (18)
C13—O1—Zn1	139.02 (14)	C11—C10—C9	118.97 (18)
C10—O3—H3A	109.5	C12—C11—C10	120.08 (17)
C18—N1—C14	118.4 (2)	C12—C11—H11	120.0
C18—N1—Zn1	129.27 (17)	C10—C11—H11	120.0
C14—N1—Zn1	112.17 (14)	C11—C12—C7	121.96 (18)
C2—C1—C6	118.32 (17)	C11—C12—H12	119.0
C2—C1—C13	120.73 (17)	C7—C12—H12	119.0
C6—C1—C13	120.93 (18)	O2—C13—O1	125.17 (18)
C3—C2—C1	120.62 (17)	O2—C13—C1	119.56 (18)
C3—C2—H2	119.7	O1—C13—C1	115.27 (17)
C1—C2—H2	119.7	N1—C14—C15	123.1 (2)
C2—C3—C4	121.40 (19)	N1—C14—C14^i^	117.24 (12)
C2—C3—H3	119.3	C15—C14—C14^i^	119.70 (15)
C4—C3—H3	119.3	C16—C15—C14	116.7 (2)
C5—C4—C3	117.49 (17)	C16—C15—C19	124.5 (2)
C5—C4—C7	120.96 (16)	C14—C15—C19	118.8 (3)
C3—C4—C7	121.54 (17)	C17—C16—C15	119.8 (2)
C6—C5—C4	121.26 (17)	C17—C16—H16	120.1
C6—C5—H5	119.4	C15—C16—H16	120.1
C4—C5—H5	119.4	C16—C17—C18	120.0 (3)
C5—C6—C1	120.90 (18)	C16—C17—H17	120.0
C5—C6—H6	119.5	C18—C17—H17	120.0
C1—C6—H6	119.5	N1—C18—C17	122.0 (3)
C12—C7—C8	116.89 (17)	N1—C18—H18	119.0
C12—C7—C4	121.29 (17)	C17—C18—H18	119.0
C8—C7—C4	121.81 (17)	C19^i^—C19—C15	121.45 (16)
C9—C8—C7	121.72 (18)	C19^i^—C19—H19	119.3
C9—C8—H8	119.1	C15—C19—H19	119.3
			
O1^i^—Zn1—O1—C13	39.5 (2)	O3—C10—C11—C12	−178.50 (18)
N1^i^—Zn1—O1—C13	−172.9 (2)	C9—C10—C11—C12	2.2 (3)
N1—Zn1—O1—C13	−89.4 (3)	C10—C11—C12—C7	0.0 (3)
O1^i^—Zn1—N1—C18	−40.1 (2)	C8—C7—C12—C11	−1.7 (3)
O1—Zn1—N1—C18	93.8 (2)	C4—C7—C12—C11	177.16 (18)
N1^i^—Zn1—N1—C18	−176.7 (2)	Zn1—O1—C13—O2	31.0 (4)
O1^i^—Zn1—N1—C14	135.31 (14)	Zn1—O1—C13—C1	−149.79 (17)
O1—Zn1—N1—C14	−90.72 (16)	C2—C1—C13—O2	1.7 (3)
N1^i^—Zn1—N1—C14	−1.20 (10)	C6—C1—C13—O2	−176.6 (2)
C6—C1—C2—C3	0.7 (3)	C2—C1—C13—O1	−177.54 (19)
C13—C1—C2—C3	−177.7 (2)	C6—C1—C13—O1	4.1 (3)
C1—C2—C3—C4	−0.3 (3)	C18—N1—C14—C15	−1.2 (3)
C2—C3—C4—C5	−0.3 (3)	Zn1—N1—C14—C15	−177.23 (17)
C2—C3—C4—C7	179.28 (19)	C18—N1—C14—C14^i^	179.4 (2)
C3—C4—C5—C6	0.4 (3)	Zn1—N1—C14—C14^i^	3.4 (3)
C7—C4—C5—C6	−179.15 (19)	N1—C14—C15—C16	2.1 (3)
C4—C5—C6—C1	0.0 (3)	C14^i^—C14—C15—C16	−178.5 (2)
C2—C1—C6—C5	−0.6 (3)	N1—C14—C15—C19	−177.1 (2)
C13—C1—C6—C5	177.78 (19)	C14^i^—C14—C15—C19	2.2 (4)
C5—C4—C7—C12	−36.7 (3)	C14—C15—C16—C17	−2.0 (4)
C3—C4—C7—C12	143.7 (2)	C19—C15—C16—C17	177.2 (3)
C5—C4—C7—C8	142.1 (2)	C15—C16—C17—C18	1.1 (4)
C3—C4—C7—C8	−37.4 (3)	C14—N1—C18—C17	0.2 (3)
C12—C7—C8—C9	1.3 (3)	Zn1—N1—C18—C17	175.43 (18)
C4—C7—C8—C9	−177.63 (19)	C16—C17—C18—N1	−0.2 (4)
C7—C8—C9—C10	0.9 (3)	C16—C15—C19—C19^i^	−178.9 (3)
C8—C9—C10—O3	178.0 (2)	C14—C15—C19—C19^i^	0.2 (5)
C8—C9—C10—C11	−2.7 (3)		

Symmetry codes: (i) −*x*+1, *y*, −*z*+1/2.

### Hydrogen-bond geometry (Å, °)

**Table:**

*D*—H···*A*	*D*—H	H···*A*	*D*···*A*	*D*—H···*A*
O3—H3A···O2^ii^	0.82	1.81	2.622 (2)	173

Symmetry codes: (ii) *x*−1/2, −*y*+3/2, *z*−1/2.
